# Supplementary material for: Functional interplay between H2B ubiquitylation and H2A.Z deposition
Source: J Biol Chem. 2026 Jul 28;302(9):113377. doi: 10.1016/j.jbc.2026.113377 (PMC13521080; doi:10.1016/j.jbc.2026.113377)
Supplement: Supplementary file 1 [file mmc1.pdf]

Supporting Information

Functional interplay between H2B ubiquitylation and H2A.Z deposition

Authors:

Younus A. Bhat, Ivano Mocavini, Erin O'Donnell, Song Tan, Oliver J. Rando, and Craig L.

Peterson

Included are Figures S1, S2, and S3
Table S1 is included as an Excel file. Table S1 shows the lists of early and late replicating genes. A list of 85 early replicating and 85 late replicating genes were manually curated based on their location within 1-2 kb of efficiently firing early or late origins.

Figure S1. Bhat et al.

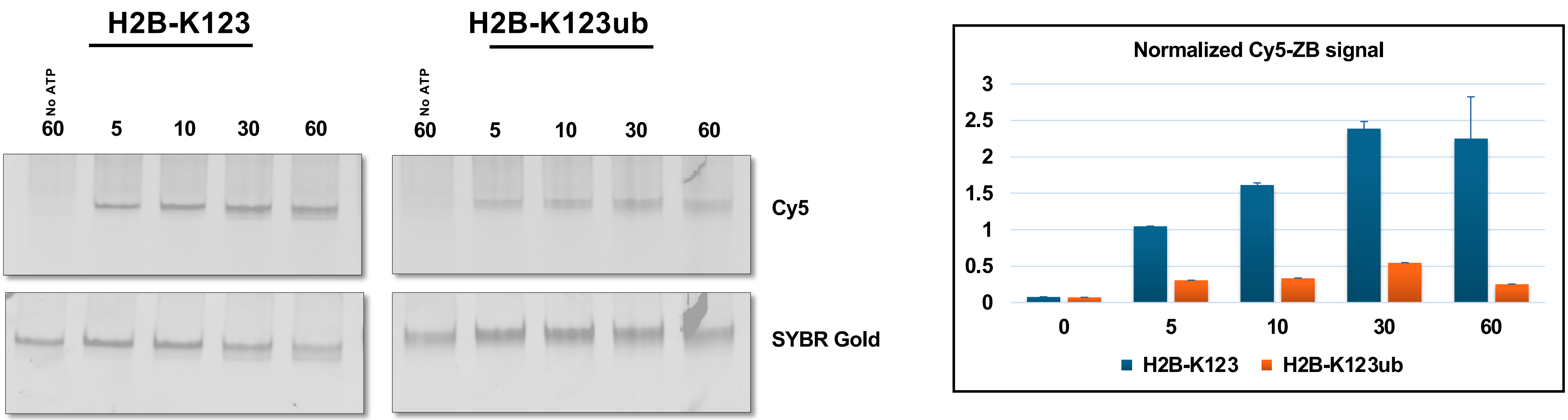

Figure S1. H2B-K123ub inhibits SWR1C-mediated dimer exchange activity. Left panels. Upper panels show dimer exchange activity using Cy5-labelled dimer and H2B-K123 and H2B-K123ub nucleosomes. Lower panels show same gels stained with SYBR gold and imaged with Typhoon imager. Right panel. Quantification of right panels. We note that this assay is prone to nonspecific, ATP-independent association of Cy5-labelled dimers with nucleosomes, leading to background signals.

Figure S2. Bhat et al.

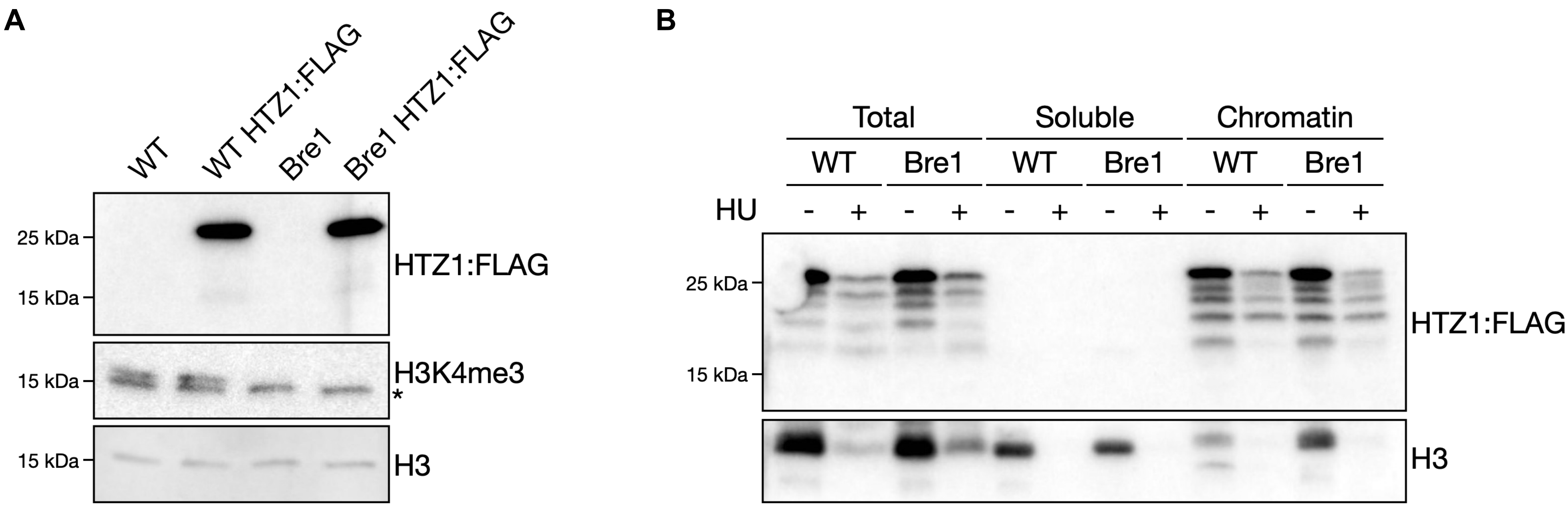

Figure S2. Bulk levels of Htz1 are not altered in bre1   cells. a) Western blot of histone extracts from WT and bre1   cells expressing or not the Htz1:FLAG construct. The asterisk in the H3-K4me3 blot indicates the band corresponding to H3; b) Western blot of total, soluble, and chromatin fractions of WT HTZ1:FLAG and bre1   HTZ1:FLAG cells upon incubation with or without HU.

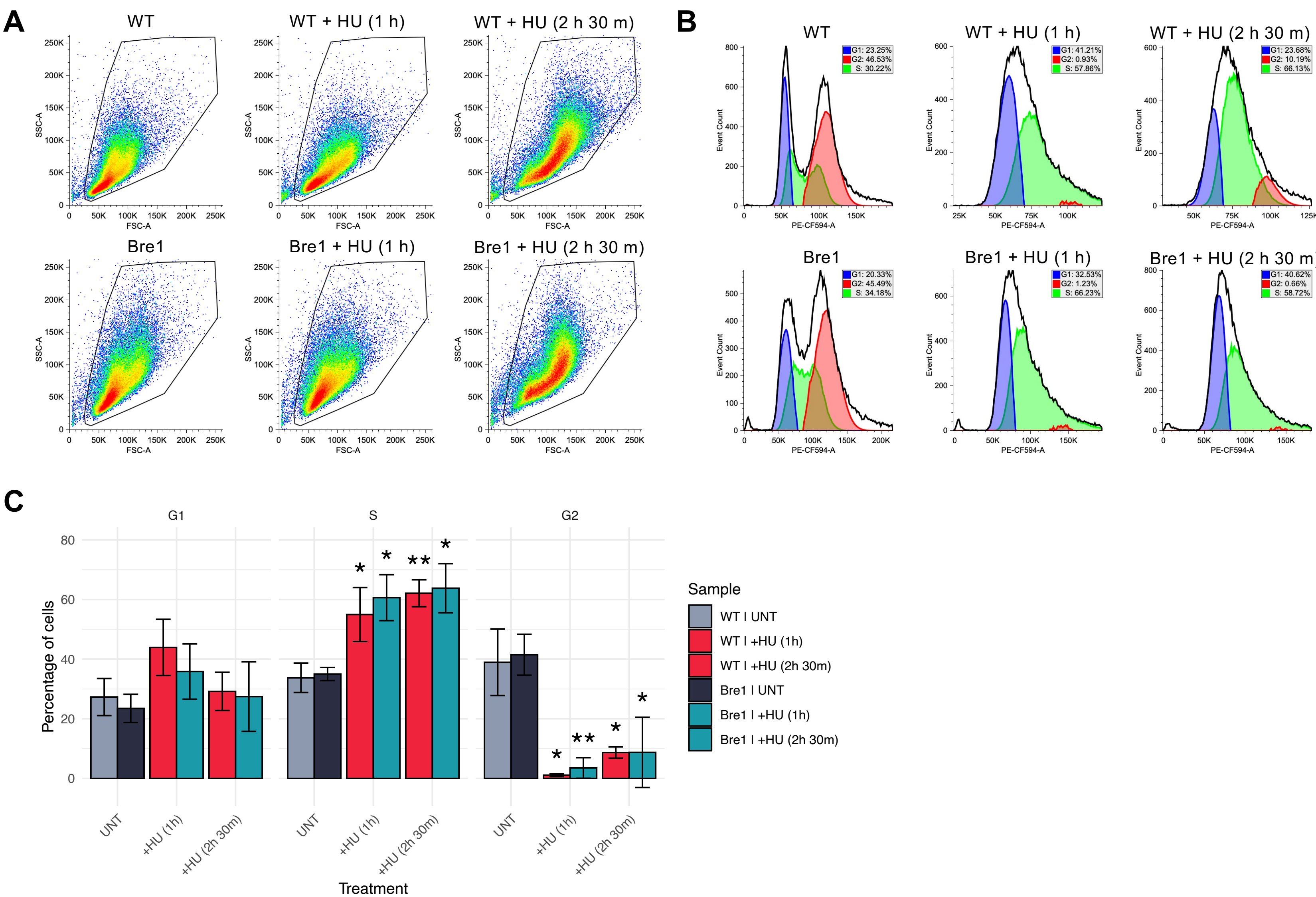

Figure S3.
Bhat et al.

Figure S3. HU induces cell cycle arrest in S-phase. a) Representative example of a FACS plot of the forward and side scatter of WT and bre1 cells upon HU treatment; b) Representative example of PE-CF594-A signal intensity, corresponding to incorporated propidium iodide, the blue, red and green shades below the curve indicate the modelled cell cycle phases (G1, G2, and S phase, respectively); c) Quantification of the percentage of cells in each phase of the cell cycle upon HU treatment, as shown in (b). Each bar and error bar represent the mean and standard deviation of three independent biological replicates; Statistical significance of the effect of HU treatment to each phase of the cell cycle was assessed using the t-test (* $p < 0.05$, ** $p < 0.01$).
